# Essential Oils of Oregano: Biological Activity beyond Their Antimicrobial Properties

**DOI:** 10.3390/molecules22060989

**Published:** 2017-06-14

**Authors:** Nayely Leyva-López, Erick P. Gutiérrez-Grijalva, Gabriela Vazquez-Olivo, J. Basilio Heredia

**Affiliations:** Centro de Investigación en Alimentación y Desarrollo A.C., Carretera a El Dorado km 5.5 Col. El Diez C.P., Culiacán, Sinaloa 80129, Mexico; nayely061005@gmail.com (N.L.-L.); erickpaulggrijalva@gmail.com (E.P.G.-G.); vazquezgabriela18@gmail.com (G.V.-O.)

**Keywords:** oregano species, terpenoids, antioxidant, anti-inflammatory, antidiabetic

## Abstract

Essential oils of oregano are widely recognized for their antimicrobial activity, as well as their antiviral and antifungal properties. Nevertheless, recent investigations have demonstrated that these compounds are also potent antioxidant, anti-inflammatory, antidiabetic and cancer suppressor agents. These properties of oregano essential oils are of potential interest to the food, cosmetic and pharmaceutical industries. The aim of this manuscript is to review the latest evidence regarding essential oils of oregano and their beneficial effects on health.

## 1. Introduction

According to the *Encyclopedic Dictionary of Polymers*, essential oils (EOs) are “volatile oils or essences derived from vegetation and characterized by distinctive odors and a substantial measure of resistance to hydrolysis” [1]. In general, EOs are complex mixtures of volatile compounds that are present in aromatic plants [2,3,4]. These compounds can be isolated from distinct anatomic parts of the plants mainly by distillation and pressing [5,6]. The main components in EOs are terpenes, but aldehydes, alcohols and esters are also present as minor components [7]. EOs are synthetized by plants in order to protect themselves from pests and microorganisms, to attract pollinating insects and for signaling processes, but recent studies have demonstrated that EOs might have beneficial effects on human health [6,7,8,9,10,11,12].

A great variety of plants are known and valued for their EO content. Oregano species are among the most widely used. Oregano is the name used to refer to a great variety of plants that share a particular flavor and odor [13]. At least 61 species and 17 genera belonging to six different botanical families are known as oregano. Verbenaceae and Lamiaceae are the most conspicuous families due to their economic importance. Within the Lamiaceae family are the plants belonging to the genera *Origanum* and *Hedeoma*; while the genera *Lippia* and *Lantana* belong to the Verbenaceae family. The other families are Rubiaceae, Apiaceae and Asteraceae [4,14]. *Hedeoma patens*, *Lippia graveolens*, *Lippia palmeri*, *Lippia alba*, *Origanum dictamnus*, *Origanum hirtum*, *Origanum onites*, *Origanum vulgare* are some examples of oregano species producing EOs [4,15,16,17,18].

EOs have great importance for the pharmaceutical, food and cosmetic industries [4]. Furthermore, there is evidence establishing that essential oils of oregano (EOO) might exert positive effects on human health. Therefore, the present manuscript will review recent investigations regarding the composition of essential oils of different oregano species and their biological activity, such as antioxidant, anti-inflammatory, anti-diabetic and antiproliferative properties.

## 2. Essential Oils Composition of Oregano Species

**Figure 1:**
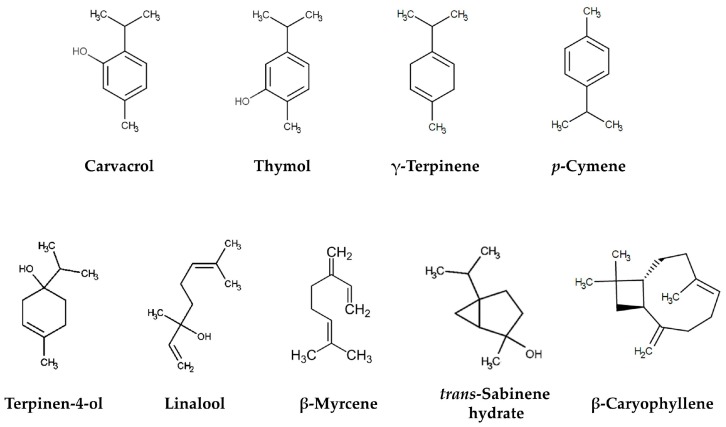
Some of the major constituents of the essential oils of oregano.

The composition of EOO has been extensively studied. EOO are very complex mixtures of compounds, in which the major constituents are terpenes, generally mono- and sesquiterpenes. The principal terpenes identified in the different species of oregano are carvacrol, thymol, γ-terpinene and *p*-cymene; while terpinen-4-ol, linalool, β-myrcene, *trans*-sabinene hydrate, and β-caryophyllene are also present (Figure 1). The proportion of these and other components in the EOO within the same species defines the chemotype. Generally, the chemotype is named after the major component of the EO, e.g., carvacrol, thymol, β-citronellol, 1,8-cineole, etc. For example, Lukas et al. [19] defined three chemotypes of *O. vulgare* according to the proportion of cymyl-compounds, sabinyl-compounds and linalool/linalyl acetate in extracts of 502 individual plants from 17 countries and 51 populations. On the other hand, González-Fuentes et al. [20] reported two chemotypes of *L. graveolens* collected from two different regions. These authors found one chemotype rich in carvacrol and thymol (wild type), while the other one (a cultivated type) showed less content of these terpenes.

It is also important to mention that the constituents and concentration of the compounds of the EOO usually vary due to a great diversity of factors such as species, pests, soil conditions, harvest season, geographical location, climatic and growth conditions [21,22,23,24,25,26,27,28,29]. The drying method of the oregano, the extraction technique and the anatomical part of the plant used for extraction also influence the EO yield and composition. For instance, Novak et al. [30] studied the influence of drying temperature on the content of EOs in *O. vulgare* ssp. *hirtum*. Natural (≈22 °C) and thermostated drying (30, 40 and 45 °C) were the temperatures evaluated. It was found that by applying 40 °C or 45 °C during the drying process a higher amount of EO can be obtained, 4.96 and 5.09 mL/100 g, respectively, than when using natural drying (4.46 mL/100 g) or 30 °C (4.16 mL/100 g). A significant reduction of the content of EOO was expected when using higher drying temperatures; nevertheless, the authors mentioned that they used relatively low temperatures to avoid the loss of EOs. On the other hand, Figiel et al. [31] evaluated the effect of different drying methods on the concentration of EOs of Polish *O. vulgare* plants. The methods tested were convective drying at 60 °C, vacuum-microwave drying (360 W) and a combination of convective pre-drying and vacuum-microwave finish-drying. The total of EOs concentration extracted from fresh oregano (33.0 g/kg) decreased significantly regardless the method used. However, the loss of EOO was less when vacuum-microwave drying method was used (27.9 mg/kg), when compared with the convective (10.2 g/kg) and the combined drying (13.1 g/kg). The authors referred to exposition of volatile compounds to hot air for long periods, the oxidation of these compounds due to oxygen presence and the interactions of volatiles with water as the main factors affecting the loss of EOs in *O. vulgare*. Authors also mentioned that due to concentration of EOs is affected by drying method so is the aroma quality of the dried sample. Therefore, vacuum-microwave drying is recommended in *O. vulgare* in order to preserve the characteristic fresh and green notes of this oregano. In a more recent study Calín-Sánchez et al. [32] evaluated the effect of the three drying methods (convective drying, vacuum-microwave drying and a combination of convective pre-drying and vacuum-microwave finish-drying) on volatile compounds of *O. majorana*. The total of EOs concentration extracted from fresh oregano (33.0 g/kg) decreased significantly regardless the method used. However, the loss of EOO was less when vacuum-microwave drying method was used (27.9 mg/kg) when compared with the convective (10.2 g/kg) and the combined drying (13.1 g/kg). The authors recommend using vacuum-drying at 240 W or a combination of convective pre-drying (50 °C) and vacuum-microwave finish-drying (240 W) so oregano can conserve the EOs content and the aroma properties. Regarding the extraction technique, Sözmen et al. [33] examined the chemical composition of EOs obtained from *O. bilgeri* by two extracting techniques: hydrodistillation and solvent-free microwave extraction. The yield of EOs extracted was higher when using microwave extraction (0.57%) than when hydrodistillation was used (0.54%). The content of carvacrol in EOs of *O. bilgeri* was 90.2% when solvent-free extraction was used, whilst when using hydrodistillation the content of this monoterpene was 84.3%. On the other hand, the content of *p*-cymene was higher when extracted by hydrodistillation (5.85%) than when solvent-free extraction was used (3.40%). The authors mention that EOs isolated from *O. bilgeri* by solvent-free microwave extraction provides advantages over the hydrodistillation technique, which are a higher extraction yield, shorter extraction time and higher content of carvacrol. Other studies about the effect of the extraction conditions or techniques on yield, composition and biological activity of EOO are those made by Hashemi et al. [34], Zheljazkov et al. [35] and Stamenic et al. [36]. In regard to the part of the plant, Han et al. [37] studied the chemical composition of EOs obtained from leaves-flowers, stems and roots of *O. vulgare* L. A total of 37 compounds were identified in the leaf-flower oils, eleven in the stem oils and 29 in the root oils. Carvacrol, thymol and caryophylline were found in the EOs of the three parts of the oregano, nevertheless their content was different. Besides, *p*-cymene and β-caryophyllene were found in the leaf-flower; while spathulenol was identified in the stem EOs and valencene in the roots.

The composition of EOO has been vastly studied. Examples of the major components of EOO are shown in Table 1.

As mentioned above, several factors affect the composition of EOs of the oregano species. As the chemical composition of the EOO is directly related with their biological activities, the next section of this review will be focused on such activities.

## 3. Biological Activities of Essential Oils of Oregano Species

Several studies have been conducted in order to determine and to evaluate the biological properties of EOO. Most of the studies are focused on the antimicrobial activity, such as antifungal, bactericidal and antiviral; nevertheless, recently other properties of these compounds have come to the attention of scientists. In this context, the next section of this review will be focused in mention current studies about bioactivities of EOO, namely antioxidant, antiproliferative and anti-inflammatory, among others.

### 3.1. Antimicrobial Effect of Essential Oils of Oregano

The EOs from herbs and spices have been extensively studied due to their anti-pathogenic properties. Numerous in vitro and in vivo researches have been conducted to evaluate the potential antibacterial, antiviral and antifungal activities of EOs [87,88]. This kind of studies are of great importance due to the emergence of antibiotic resistant strains, the increase in the population with lower immunity and increased incidences of drug resistant biofilm associated infections. These studies have focused on individual as well as a combination of EOs, or by extracting by several methods the EOs from different herbs and spices. Different species of oregano are among the most studied herbs and different composition in EOs have been used for this purpose, nonetheless the most common compounds found in oregano are thymol and carvacrol [88,89].

EOO have been studied as anti-pathogenic agents in meat products and post-harvest fruits and vegetables. They have sometimes been used in combination with edible coatings such as the case when used in post-harvest fruits and vegetables. Precautions are taken in the possible modification of the organoleptic properties of such products when added with essential oils. On the other hand, in some cases, the antioxidant (AOX) capacity of the final product is increased which is seen as a positive side effect of the use of these compounds [90,91,92,93].

It is important to mention that the aim of this review is not to summarize the recent findings on this subject, but to mention research on other biological properties of EOO. Nonetheless, we consider of relevance to recommend the studies by Adame-Gallegos et al. [88] and Rodriguez-Garcia et al. [87] for further information.

### 3.2. Essential Oils of Oregano as Antioxidants

Oxidative stress is the disturbance in the pro-oxidant-antioxidant balance in favor of the former, leading to potential damage; and is often caused by the attack of reactive species, such as hydrogen peroxide (H_2_O_2_), superoxide anion (O_2_**^•^**^−^), hydroxyl (HO**^•^**), peroxyl (RO_2_**^•^**), and alkoxyl (RO**^•^**) radicals, upon the constituents of living organisms [94,95]. Oxidative stress can cause an increasing in cell proliferation, adaptation by up-regulation of defense systems, DNA damage, senescence and cell death. Consequently, many studies have positively correlated oxidative stress with the pathogenesis of diseases, such as Alzheimer’s disease, Parkinson’s disease, chronic-inflammation, arthritis, some types of cancer, diabetes and atherosclerosis, among others [94,95,96,97]. Several phytochemicals have been studied for their potential scavenging activity towards reactive species and capacity to neutralize oxidative stress. EOs are among the targeted phytochemicals due to their AOX capacity since it is of great importance due to their potential to prevent oxidative damage [95,98,99]. The AOX properties of EOs from different species of oregano have been studied; and it is worth to mention that some of the EOs (carvacrol, thymol) that have been studied in other plant species for their AOX properties can also be found in different oregano species. Nonetheless, this does not guarantee their capacity to neutralize oxidative stress and to prevent oxidative damage, since the differences in the composition of EOO can modify the biological activity [16,57,67,100,101].

When EOs of *O. vulgare* subsp. *hirtum* were supplemented to sows, the oxidative stress markers were reduced in their piglets, showing lower serum levels of 8-hydroxy-deoxyguanosine and thiobarbituric acid reactive substances; which causes severe DNA damage [102]. It has been stated that some antioxidants can act as both pro-oxidant and AOX depending on the dose [103,104]. In this sense, carvacrol and thymol, monoterpenes found in oregano, may induce oxidative stress in several Caco-2 cells when used in high concentrations (>230 μM), since these terpenes might increase the level of reactive oxygen species and decrease the content of glutathione [105]. Numerous studies have shown the AOX properties of EOO [57,83,90,100,106,107,108]; however, further pre-clinical and clinical studies are needed with special focus on their possible toxic effects in order to establish safe doses.

Beside the benefit of EOO in health, there have been efforts to study the AOX activity of these compounds to use them as food preservatives [109,110]. Different studies have shown that the AOX effects of EOO are related to the presence of phenolic structures such as thymol and carvacrol [55] and so these compounds could replace synthetic antioxidants currently used in the food industry, and due to their natural origin improve health. In this regard, the half maximal inhibitory 2,2-diphenyl-1-picrylhydra-zyl (DPPH) radical concentration (IC_50_) values of EOs in different parts of *O. vulgare* (leaf-flowers, roots and stems) varied from 0.332 to 0.501 mg/mL. Although, a great AOX activity was observed in EOO, it was lower than the butylated hydroxytoluene (BHT, IC_50_ = 0.161 mg/mL). In fact, leaf-flower oils showed the best AOX activity [37]. In the 2,2′-azino-bis(3-ethylbenzothiazoline-6-sulphonic acid) (ABTS) assay, EOs of *O. compactum* showed comparable AOX activity (IC_50_ = 2.0 mg/L) to Vitamin C (IC_50_ = 1.9 mg/L). It is believed that a possible synergy between the components of EOO can explain their AOX potential [111]. The EOs of *O. vulgare* L. have scavenging activity for the DPPH radical (IC_50_ = 0.2 µg/mL) when compared to the synthetic antioxidant BHT. Likewise, oregano showed an IC_50_ = 0.12 µg/mL in the OH radical scavenging activity assay. The main components of EOO were thymol and carvacrol. In this study the antiradical activity of the isolated compounds, thymol and carvacrol, was also assessed. It was found that synergic effect of the main components reported in EOO is likely to be responsible for the radical scavenging effect [110]. By comparing the AOX activity of EOs of *O. vulgare* with the synthetic antioxidant ionol measured by the DPPH assay, Alinkina et al. [112] reported an antiradical efficiency slightly smaller than ionol; the EOs where mainly constituted by carvacrol and thymol.

Differences in the AOX activity and chemical composition of EOO have been reported with relation to the geographic origin, the mode of extraction or plant different phenological stage [73]. Asensio et al. [22] assessed the AOX activity of EOs of four different types of oregano from central and southern regions of Argentina. The oregano species evaluated were *O*. x *majoricum*, *O. vulgare* ssp. *vulgare* and ssp. *hirtum*. The AOX activity was measured by the ABTS assay. The values ranged from 0.234 to 0.163 mM Trolox/mg of EOs. In the same study, the authors reported high amounts of δ- and γ-terpinene and oxygenated monoterpenes such as terpinen-4-ol and thymol, suggesting that these compounds are the main contributors to the Trolox equivalent antioxidant capacity (TEAC) values. In addition, the ferric reducing antioxidant power (FRAP) value of oregano varied from 0.184 to 0.072 mM/mg. Besides, the oregano samples showed a bleaching inhibition of up to 89.2% (20 µg/mL of EOs) in the β-carotene assay. These authors also report the oxygen radical absorbance capacity (ORAC) value in a range from 1.708 to 1.024 trolox equivalents [22]. On the other hand, EOs of 51 wild plants of *Origanum* ssp. collected from different areas of Sicily showed a variation in the concentration that inhibits 50% (IC_50_) of the UV radiation-induced peroxidation in liposomal membranes (UV-IP test). The values were in a range from 3.81 to 11.05 µg/mL. EOO also showed a good radical scavenging activity measured by Folin-Ciocalteau in a range of 243.92 to 413.36 mg/mL. This was attributed to the occurrence of phenolic structures like thymol and carvacrol as well as the presence of monoterpene hydrocarbons such as γ-terpinene [101]. Furthermore, Quiroga et al. [113] evaluated the DPPH inhibition percentages of four oregano EOs from Argentina (*O. vulgare* ssp. *vulgare*, *vulgare* ssp. *virens*, *O.* x *appli*, *O*. x *majoricum*). The oregano species with the higher content of thymol, *O. vulgare* ssp. *virens* (29.7%) and ssp. *vulgare* (26.6%), were also the ones with the best radical scavenging activity (0.98 to 0.90 µg/mL respectively). The authors mention that these EOO might be used to replace synthetic antioxidants on food products with high content of lipids. On the other hand, it has been demonstrated that EOs obtained from *O. glandulosum* from different locations of Tunisia have low AOX activity when compared to ascorbic acid. The IC_50_ for the EOO value varied in a ranged from 59.2 to 79.8 mg/L. The authors suggest that *O. glandulosum* EOs are potent natural antioxidants and that might decrease the degree of lipid peroxidation [72].

Traditionally, EOO are extracted by hydrodistillation. Nonetheless, different methods of extraction might have an effect on the AOX activity of EOO since this could affect the composition of the EOs. For example, fractions of EOO (*Origanum vulgare* L.) were obtained by short path molecular distillation. The AOX activity, measured by the DPPH assay, was different between the residues and the distillates obtained. Even though all fractions were mainly composed by γ-terpinene, terpineol-4-ol, carvacrol and α-terpinene, these compounds were found in different proportions. Carvacrol, a phenolic-monoterpene, was the most concentrated compound in the residues, which might be responsible for their higher AOX activity [56]. Similarly, Borgarello et al. [114] obtained fractions of EOs of *O. vulgare* ssp. *hirtum* by molecular distillation and evaluated their AOX activity by the DPPH assay. The residues obtained showed lower IC_50_ values (1.0–1.6 mg/mL) than the EOs (0.45 mg/mL), which indicates an increase in their AOX properties. Residues obtained were concentrated in thymol, suggesting that the free radical scavenging capacity might be due to this phenolic monoterpene [114]. Furthermore, EOs of *O. hirtum* also showed important AOX activity by ABTS assay [80].

The effect of distillation time on the AOX activity, assessed by the ORAC method, of EOs of *O. vulgare* L. was studied by Zheljazkov et al. [35]. The authors determined that in spite of the time of hydrodistillation modified the composition of the EOs, it did not affect the AOX activity. Even though the values of ORAC were different at 20, 80 and 360 min of hydrodistillation (60.8, 75.4 and 60.4 μM trolox equivalents/g, respectively) these differences were not significant, which might indicate that the AOX of EOs were not converted or lost during the distillation process [35]. The AOX activity of EOs of *O. vulgare* ssp. *hirtum* was also measured [115]. The inhibition on ABTS radical of EOO was similar when obtained by solvent-free microwave extraction (99.09–94.77%) and conventional hydrodistillation (97.45%). Nevertheless, when the EOs were obtained by supercritical fluid extraction the inhibition on ABTS was 30.45%. The authors mention that this may be due to the difference in the composition of essential oil [115].

The harvesting time might also influence the antioxidant activity of EOO. For instance, Mechergui et al. [73] evaluated the AOX activity, using the DPPH assay, of EOs obtained from leaves and flowers of *O. glandulosum*. It was demonstrated that AOX activity differs depending on the harvest year and location of oregano. The antiradical activity (expressed as IC_50_) varied from 59.2 mg/L in 2007 to 226.19 mg/L in 2008 and 143.91 mg/L in 2009 for oregano collected in Nefza, and from 79.8 mg/L in 2007 to 137.66 mg/L in 2008 and 151.85 mg/L in 2009 for Krib population. The differences in the concentration and composition of *p*-cymene, γ-terpinene, thymol and carvacrol in the EOs might be responsible for the AOX activity changes [73]. Likewise, the free radical scavenging activity, measured by the DPPH assay, of *O. onites* harvested at different times was affected, the samples obtained in June showed lower IC_50_ values (116.74 μg/mL) and higher AOX capacity than those obtained in July (123.75 μg/mL), August (128.86 μg/mL) and September (132.93 μg/mL). Similarly, the oregano collected in July showed higher AOX activity than the ones collected in the following months [50].

EOO have demonstrated to have efficacy as antioxidants and might have the potential for delaying lipidic oxidation. This could give to the EOO the capacity for inhibiting the cell damage caused by free radicals [116]. Furthermore, EOO prevented autoxidation of polyunsaturated fatty acid esters isolated from mouse brain, being carvacrol and thymol the antioxidant components [106]. The AOX activity of *O. vulgare* L. from Argentina was measured by the Rancimat method and DPPH assay. EOs of *O. vulgare* showed an antioxidant activity index (AAI) of 1.20 and a strong radical scavenging activity (IC_50_ 0.357 µg/mL). This was attributed to the phenol-type constituents of the EOO (thymol and carvacrol) [57]. The AOX activity of the EOs of *O. syriacum* from Egypt was assessed by Viuda-Martos et al. [52]. The main components in the oils were thymol (21.04%) and γ-terpinene (18.96%). The effective concentration of the EOO required to scavenge DPPH radical was 50 g/L and the scavenging values expressed as inhibition percentage was 87.23%. The AOX activity of EOO (IC_50_ = 6.66 g/L) was lower than the positive controls, the synthetic antioxidants BHT (IC_50_ = 0.53 g/L) and ascorbic acid (IC_50_ = 0.42 g/L). In TBARs assay, EOO showed the highest percentage of inhibition with 85.79% at 50 g/L with an EC_50_ of 3.99 g/L, as compared to the EOs of other herbs analyzed, and did not showed statistical difference with the positive control (ascorbic acid, EC_50_ of 3.70 g/L). Besides, EOO were capable of chelating iron(II). By the Rancimat method, EOO showed AOX activity but lower than that of synthetic antioxidants. The authors consider that the EOs of O. syriacum are good antioxidant compounds and have potential application as food preservatives [52]. On the other hand, EOs from *O. dictamnus*, *O. libanoticum* and *O. microphyllum* exhibited a poor antiradical activity compared to BHT. However, in FRAP assay, all EOs showed significant antioxidant activity. The main compound was *p*-cymene (32.7%). It is stated by the authors that EOs of this specific species are not active in catching free radicals but they have a major ferric reducing/antioxidant power [117]. There are several other studies that have reported the AOX activity of EOs of different species of oregano [42,55,83,85,118].

The AOX properties of EOs are thought to be related, at least in part, with the mechanisms in which they exert their effect on the oxidative stress. Some reported mechanisms are: (i) free radical scavenging activity, (ii) modulation of AOX enzymes (superoxide dismutase); and (iii) inhibition of pro-oxidation. In this sense, EOs are effective free radical scavengers as reported through different and numerous in vitro assays such as FRAP, iron (III and II) reducing power, DPPH and ORAC [67,99]. The AOX properties of EOs are suggested to be related with their multiple bonds, the higher the length of the conjugated terpenes, the greater the scavenging activity [99]. Because of the radical scavenging ability of the EOO, it has been reported that these compounds can be used as a health promoting substances in the prevention of chronic diseases and neurodegenerative disorders, which are linked to oxidative stress [12,83,107,119]. Antioxidant properties from EOO offers the possibility of using these oils as preservatives and flavor for food or nutraceutical products [101].

### 3.3. Anti-Inflammatory Activity of Essential Oils of Oregano Species

Inflammation is a normal biological response of the body to tissue damage, infections and chemical or physical agents [120]. During inflammation the production of inflammatory mediators is triggered. Examples of these mediators are cytokines, prostaglandins, enzymes, nitric oxide (NO) and reactive oxygen species (ROS), among others [121]. If inflammation is not controlled the pro-inflammatory mediators are overproduced which might induce pathologic processes related to diseases such as arthritis, atherosclerosis and cancer to name a few [122,123]. Consequently, inhibition of the mediators is an imperative goal to treat inflammatory diseases.

There is evidence that mention that EOO have the ability to exert anti-inflammatory activity. For example, Leyva-López et al. [16] demonstrated that terpenes, such as thymol and carvacrol acetate, obtained from the three Mexican oregano species, *L. graveolens*, *L. palmeri* and *H. patens* reduced significantly the levels of ROS and NO produced by RAW 264.7 macrophage cells stimulated with lipopolysaccharide (LPS). Furthermore, EOs of *O. majorana* (10 μg/mL) reduced the production of tumor necrosis factor-alpha (TNF-α), interleukin-1β (IL-1β) and IL-6 in LPS-activated THP-1 human macrophage cells [124]. Recently, Han and Parker [125] showed that EOs obtained from *O. vulgare* significantly inhibited the levels of the inflammatory biomarkers monocyte chemoattractan protein-1 (MCP-1), the vascular cell adhesion molecule-1 (VCAM-1) and the intracellular cell adhesion molecule-1 (ICAM-1) on activated-primary human neonatal fibroblasts. These findings suggest that the EOO have anti-inflammatory properties.

The individual components of EOs of oregano have also been studied to better understand their effect on inflammation. For example, Lima et al. [126] demonstrated that carvacrol exerts anti-inflammatory activity on a typical mice inflammation model. When carvacrol was administrated to mice (at 50 and 100 mg/kg), presenting paw edema, the levels of IL-1β and prostaglandin E_2_ (PGE_2_) prostaglandins were diminished. The reduction on the mRNA expression of IL-1β and cyclooxygenase-2 (COX-2) might be responsible for the effects mentioned. On the other hand, the levels of the cytokine IL-10 in the swollen paw were improved by carvacrol. So, the anti-inflammatory effect of carvacrol is due to the reduction of pro-inflammatory mediators but also to the increasing of anti-inflammatory cytokines (IL-10) [126]. Carvacrol has also showed the ability to prevent obesity in mice by modulating expression of genes involved in inflammation [127] and to attenuate induced liver injury and ulcer in rats [128,129]. Other EOs components, such as *p*-cymene [130] and β-caryophyllene [131,132,133,134] have also demonstrated anti-inflammatory properties.

The studies mentioned above indicate that diverse oregano species might be used as anti-inflammatory agents and could be used in formulations for the prevention or the treatment of inflammation-related diseases. Nevertheless, and since the EOO might exert toxic effect on cells, several in vivo and clinical studies are needed before the EOs can be used as an alternative to treat inflammation.

### 3.4. Essential Oils of Oregano Species and Cardiovascular Diseases

Cardiovascular diseases (CVD) are the major cause of death in several countries and are increasing worldwide. CVD is a chronic inflammatory condition that is accelerated by various factors such as smoking, diabetes and inflammation [135,136]. The major cause of CVD is atherosclerosis, which is the result of an abnormal inflammatory response [122,137]; as some pro-inflammatory cytokines IL-1β and TNF-α are involved in the process of leukocyte adherence [136,138].

As previously mentioned, EOs are natural compounds that have been studied for their biological activities such as anti-inflammatory and cardiovascular effects. On this matter, research on the effects on anti-CVDs of EOs have focused on individual components of essential oils such as carvacrol, thymol, eugenol, and γ-terpinene from several sources. Interestingly, these studies have shown that some EOs can lower total plasma cholesterol and triglyceride levels, which contribute to the formation of atherosclerosis. On the other hand, studies using essential oil extracts from herbs such as oregano are scarce [11,139,140].

While most studies are focused on the potential beneficial effects of individual EOs, or the combination of them, it is important to mention that some efforts have been extended on the study of the bioactive properties of EOs from different species of oregano. Among the most notorious studies reported to date, anti-inflammatory properties of different species of oregano have been reported [16,140]. Nonetheless, given the relationship between inflammation and the development of CVD, this could be a way in which EOO may exert an indirect cardioprotective effect; yet there is a lack of studies and therefore the precise mechanism in which this occur is still not fully known.

A study by Ocaña-Fuentes et al. [140] used oxidized-LDLs activated THP-1 macrophages treated with EOO from *O. vulgare*, which main compounds were sabinene hydrate, thymol and carvacrol. The authors reported a decrease in the synthesis of pro-inflammatory TNF-α, IL-1β and IL-6 cytokines, as well as an increase in the production of anti-inflammatory cytokine IL-10. These results are important because it shows that EOs from oregano may be used in novel treatments of inflammation-related chronic diseases such as atherosclerosis.

It is important to mention that the mode of action of EOO on their effect on cardiovascular health is not fully known due to the lack of studies. However, Dantas et al. [139] suggest the possible anti-hypertensive properties, of EOs such as carvacrol by acting as hypotensive agents due to the vasodilatation that involves, the inhibition of the Ca^+2^ influx through Ca_v_ and TRP channels. In this regard, TRP channels are a superfamily of receptors that have been suggested to be involved in the development of cardiac hypertrophy, heart failure, cardiac arrhythmias, vascular remodeling and pulmonary hypertension [141].

Hypertension is the most common chronic disease and a major risk factor for morbidity and mortality. Among the various factors associated in the pathophysiology of hypertension are the alterations in cellular cations, such as sodium (Na^+^), calcium (Ca^2+^), potassium (K^+^) and magnesium (Mg^2+^), all of which are known to increase systolic blood pressure. In this regard, EOs have exhibited modulatory properties on various ion channels, nonetheless, studies on EOs from oregano are scarce [142].

It has been observed that some of the EOs are able to significantly promote the cardiovascular system and can lead to vasorelaxation, hypotension and regression of atherosclerosis process. Many studies have been conducted to evaluate the role of EOs on the vasodilation and the heart rate. To a better understanding of the potential protective effect on cardiovascular health of EOs the studies by Saljoughian et al. [143], Alves-Silva et al. [144] and Oz et al. [142] should be consulted.

### 3.5. Essential Oils of Oregano and Their Effect on Metabolic Syndrome

Obesity and metabolic syndrome are life-threatening events that lead to growing health issues worldwide. Obesity is the central and causal component in metabolic syndromes, resulting from an imbalance between energy intake and energy expenditure that can cause impaired glucose tolerance, insulin resistance, and type 2 diabetes [98,145]. In particular, metabolic syndrome is defined by the International Diabetes Federation as a cluster of the most dangerous heart attack risk factors: diabetes and raised fasting plasma glucose, abdominal obesity, high cholesterol and high blood pressure [145,146].

Although a healthy lifestyle and diet control are among the prevention strategies of metabolic syndrome, drug therapy is also used in the treatment of abdominal obesity, raised fasting plasma glucose and diabetes. Among the most common drugs used are metformin, which is a well-known adenosine monophosphate-activated protein kinase (AMPK) activator; and the inhibitors of α-glucosidase and α-amylase, which are the main enzymes that mediate the metabolism of dietary carbohydrates. Nonetheless, such inhibitors can often cause secondary unwanted effects such as abdominal discomfort, therefore efforts are extended in searching for new natural alternatives without adverse effects [147,148,149,150].

As a result, research has been extended on the potential anti-diabetic properties of phytochemicals, such as polyphenols and EOs, from numerous sources. Research on this matter has mainly been focused on the hypoglycemic effect of polyphenols; however, the potential inhibitory effect of compounds like EOs is often neglected. Nonetheless, some studies have suggested that EOs from numerous plants may play a key role in reversing some metabolic syndrome factors. It is important to mention that some herbs are still being used in folk medicine as a primary health care in the treatment of diabetes in some developing countries. And some species of oregano are among the most studied herbs as potential hypoglycemic therapeutic agents [98,151].

The anti-diabetic properties of EOO have been related to the main components such as carvacrol and thymol [83,152]; in this sense some studies on plants belonging to the Lamiaceae family, suggest that the mode of action of EOs may vary depending on that composition [150].

With respect to the above mentioned, a study by Yen et al. [150] using 3T3L1 adipocytes analyzed the glucose consumption activity and lipid drop accumulation activity of EOs obtained from the Taiwanese market. Interestingly, they found a significant increased glucose consumption and inhibition of lipid accumulation into the 3T3-L1 adipocytes when treated with EOs from *Melissa officinalis* (a plant member of the Lamiaceae family). It is suggested that the EOs of *M. officinalis* activate the AMPK/ACC pathway, which is involved in ATP restoration and mediation of lipid metabolism via regulation of acetyl-CoA carboxylase. Similarly, Sarikurkcu et al. [83] showed that the EOs from two species of oregano (*O. vulgare* subsp. *vulgare* and subsp. *hirtum*) possess α-amylase and α-glucosidase inhibitory activity. Interestingly the major compounds identified in these species were thymol, carvacrol, *p*-cymene, γ-terpinene, and linalool. Similarly, Alef et al. [153] reported the inhibition of the amylase by oils of *O. vulgare*, and that when in combination with rosmarinic acid, the inhibition was higher, therefore suggesting that the combination of phenolic compounds with EOs from oregano may contribute to additional amylase inhibitory activity. It is important to mention that variability in the EOs composition from different species of oregano has been previously reported [16]. Similarly, Bayramoglu et al. [154] reported that whilst carvacrol from *O. onites* L. had no effect on the biochemical parameters of diabetes (serum glucose, insulin, and total cholesterol) in streptozotocin-induced diabetic rats, it did manage to reduce the streptozotocin liver damage. It is important to mention that the potential anti-diabetic properties of individual EOs have been studied, nonetheless, when in combination with other compounds this effect can be modified, therefore we encourage future research on the anti-diabetic and anti-metabolic syndrome properties EOs from different species of oregano.

### 3.6. Antiprolifertive and Citotoxic Activity of Essential Oils of Oregano

Essential oil constituents could exert antiproliferative effect. Different mechanisms such antioxidant, antimutagenic, antiproliferative, among others are responsible for their chemopreventive properties [155] The antiproliferative effects of EOs have been demonstrated in diverse cancer cell models through several pathways [156].

Marrelli et al. [117] studied the antiproliferative activity of EOs of *Origanum* species. At 100 µg/mL concentration, EOs derived from *O. dictamnus* showed the most interesting biological activity with an inhibition on colon carcinoma cell line (LoVo) of 58.39% after 24 h. Additionally, the IC_50_ value for this essential oil was 84.76 µg/mL after 24 h and 72.26 µg/mL after 48 h of treatment. In the same study, it was reported that the EOs from *O. dictamnus* and *O. libanoticum* had an antiproliferative activity of 49.83% and 48.50% in the hepatocarcinoma cell line (HepG2) at 100 µg/mL concentration. Begnini et al. [157] showed that EOs from *O. vulgare* inhibit cell proliferation in human breast adenocarcinoma (MCF-7), and human colon adenocarcinoma (HT-29) cells at 50 mg/mL (60.8% and 48.9%, respectively).However, an increase in the EOO concentration did not increase the cell growth inhibition. The authors implied that the effect could be attributed to the main components (terpinen-4-ol, thymol, γ-terpinene and carvacrol). Besides, EOs of *O. hirtum* significantly reduced the proliferation of human lung adenocarcinoma epithelial (A549) cell line, after 24 h incubation, compared with untreated control cells [76]. Furthermore, it was suggested that the EOO modifies the onset of mitosis, possibly prior to the G2 phase and prophase.

Many evidences have shown that EOO had antitumor effect. EOO have shown antitumor activity both in in vivo and in vitro assays. An in vivo study reported that low doses of EOO in a three-month period exerted preventive action by decreasing the sizes of tumors by 1.5 times in diseased animals. It was suggested that the EOO could possible affect the development and progression of the tumor process via the activation of regulator cell molecules [158]. The in vitro antitumor activity of EOs of *O. onites*, was analyzed against rat adipose tissue endothelial cells and c-H-ras transformed rat embryonic fibroblasts (5RP7) cells. EOO at 125, 250 and 500 µg/mL resulted in significant inhibition of cell viability. In addition, EOO induced apoptosis of 5RP7 cells and blocked in vitro tube formation which accounts for its angiogenic activity [159].

Besides, EOO have shown activity against genotoxic agents, which are capable of altering DNA and thereby causing cancer or mutation [160]. The effects of AFB_1_ (5 µM), a cancer promotor, on human peripheral lymphocytes, were decreased after treatment with EOs of *O. rotundifolium* (1.5 µL and 2.0 µL). This activity could be attributed in part to the EO composition and its antioxidant capacity [161]. Similarly, the combined treatment of prallethrin and EOs from *O. majorana* in bone marrow cells of rats, resulted in the reduction of the chromosomal aberration (54.54%), thus, EOO demonstrated to have genotoxic effect. The effects could be attributed to the scavenge ability of EOO and its contribution in hindering lipid peroxidation [162].

Furthermore, in human breast (MCF-7) and prostate (LNCaP) cancer cell lines, EOs of *O. majorana* and *O. vulgare* showed an inhibitory effect on cancer cell viability in a range of 79–88% at 0.5 mg/mL (24 h). *Origanum majorana* was more cytotoxic than *O. vulgare* against MCF-7 and LNCaP cells with IC50s of 70.0 and 85.3 µg/mL [163]. Similarly, cytotoxicity of EOs was tested using human keratinocyte (HaCaT) and lung cancer (A549) cell lines. Cell viability decreased in a concentration-dependent manner on both cell lines. Essential oils of *O. hirtum* exhibited cytotoxicity both on HaCaT and A549 cells with IC_50_ values ranging from 146.8 to 148.8 μg/mL and from 147.1 to 177.1 μg/mL respectively, after 24, 48 and 72 h [80]. In another study, EOs of *O. compactum* have shown to be non-toxic towards MCF-7 cells (IC50 > 100 mg/L) [111].

Essential oils from oregano have been proposed as dietary inhibitors of mutagenesis and carcinogenesis [162]. Nonetheless, investigations about anticarcinogenic effect of EOO are scarce. Likewise, there are considerably fewer works dedicated to the biological activity of EOs using cell cultures and using in vivo models [158]. Furthermore, the molecular targets at the genome level and the involved genetic pathways of the mechanisms of EOO are remaining poorly explored [164].

A brief compilation of the biological activities, mentioned above, of EOs from different species of oregano is shown in Table 2.

## 4. Conclusions

This review tries to enlighten about the therapeutic use of essential oils extracted from different oregano species. The many studies mentioned in this manuscript might be taken in consideration for those interested in searching new components or natural drugs that can be used to treat or prevent several diseases of worldwide importance, such as diabetes and cancer. Nevertheless, in spite of the many benefits of essential oils from oregano, it has been reported that they can exert adverse effects. As a consequence, pre-clinical studies are needed to ensure the security of the use of these compounds in humans. Likewise, administration strategies should be studied to enhance the effect of such compounds.

## Figures and Tables

**Table 1:** Principal components of essential oils of different oregano species.

Oregano Species	Origin	Components	Yield *	Reference
*H. patens*	Mexico	Thymol, *trans*-piperitol, carvacrol acetate, carvacrol, camphene, β-myrcene, γ-terpinene, cis-p-mentha-1(7), 8-dien-2-ol, α-muurolene, α-calacorene, bulnesol, cadalene, viridiflorol.	NR	[16]
*L. grandis*	Brazil	Carvacrol (37.12%), *p*-cymene (11.64%), thymol (7.83%), β-caryophyllene (3.93%).	2.7%	[38]
*L. graveolens*	Mexico	Thymol, carvacrol acetate, carvacrol, camphene, β-myrcene, γ-terpinene, cis-p-mentha-1(7),8-dien-2-ol, viridiflorol.	NR	[16]
*L. origanoides*	Colombia	Thymol (78.7%), *p*-cymene (6.6%), γ-terpinene (2.7%), *trans*-β-caryophyllene (2.1%).	NR	[39]
*L. palmeri*	Mexico	Thymol, α-cedrene, *trans*-piperitol, eugenol, carvacrol acetate, β-selinene, γ-cadinene, spathulenol.	NR	[16]
*O. acutidens*	Mexico	Carvacrol (8.76–24.57%), *p*-cymene (14.25–22.37%), thymol (15.11–21.39%).	5.0–6.0%	[40]
	Turkey	Carvacrol (76.21%), *p*-cymene (7.42%), borneol (3.19%), γ-terpinene (1.38%).	1.45%	[41]
	Turkey	Carvacrol (65.13%), *meta*-cymene (9.15%), *trans*-β-caryophyllene (4.43%), γ-terpinene (3.54%).	3.1%	[42]
*O.* x *applii*	Argentina	Thymol (30.77%), *trans*-sabinene hydrate (29.63%), γ-terpinene (4.4%), terpinen-4-ol (3.23%).	1.83 ± 0.27 mg/g dw	[43]
*O. ehrenbergii*	Lebanon	Carvacrol (79.0%), *p*-cymene (4.4%), carvacrol methyl ether (2.7%), γ-terpinene (2.6%).	3.19%	[44]
*O. bilgeri*	Turkey	Carvacrol (84.30–90.20%), *p*-cymene (3.40–5.85%), γ-terpinene (0.47–1.20%), thymol (0.69–1.08%).	0.54–0.57%	[33]
*O. libanoticum*	Lebanon	β-Caryophyllene (26.8%), caryophyllene oxide (22.6%), germacrene D (17.2%), thymol methyl ether (10.5%).	0.16%	[44]
*O. majorana*	Brazil	1,8-Cineole (20.9%), terpinen-4-ol(20.4%), γ-terpinene (8.5%), *p*-cymene (7.0%).	NR	[45]
*O.* x *majoricum*	Colombia	*trans*-Sabinene hydrate (14.5%), γ-terpinene (14.0%), carvacrol methyl ether (6.0%), terpinen-4-ol (6.0%).	NR	[39]
	Turkey	Limonene (88.01%), thymol (11.98%).	NR	[46]
	Argentina	*trans*-Sabinene hydrate (24.3–28.1%), thymol (12.1–17.4%), γ-terpinene (7.0–7.5%).	NR	[22]
	Argentina	*trans*-Sabinene hydrate (36.77%), thymol (17.77%), γ-terpinene (5.9%), α-terpinene (3.9%).	3.9 ± 0.25 mg/g dw	[43]
*O. hypericifolium*	Turkey	*p*-Cymene (34.33%), carvacrol (21.76%), thymol (19.54%), γ-terpinene (13.91%).	2.9%	[47]
*O. onites*	Greece	Carvacrol (79.63%), γ-terpinene (3.89%), *p*-cymene (3.51%), β-caryophyllene (2.24%).	3.62%	[18]
	Greece	Carvacrol (62.6%), *p*-cymene (8.87%), γ-terpinene (8.45%), β-myrcene (2.92%).	NR	[48]
	Greece	Carvacrol (69.0–92.6%), *p*-cymene (0.5–9.5%), γ-terpinene (0.3–7.9%), borneol (0.8–5.5%).	3.0–7.0%	[49]
	Turkey	Carvacrol (85.86%), γ-terpinene (4.43%), β-phellandrene (3.20%), *p*-cymene (1.83%).	4.7 ± 0.06%	[21]
	Turkey	Carvacrol (83.97–88.65%), thymol (0.80–7.48%), γ-terpinene (2.63–6.15%), *p*-cymene (1.52–3.16%).	2.5–3.2%	[50]
*O. syriacum*	Egypt	Carvacrol (81.38%), *p*-cymene (8.48%), γ-terpinene (1.98%), β-myrcene (1.32%).	5.5%	[51]
	Egypt	Thymol (31.73%), γ-terpinene (14.32%), linalool (9.44%), terpinen-4-ol (7.68%).	4.63%	[51]
	Egypt	Thymol (21.04%), γ-terpinene (18.96%), terpinen-4-ol (17.20%), α-terpinene (7.41%).	0.6%	[52]
	Lebanon	Carvacrol (60.8%), *p*-cymene (8.4%), thymol (7.9%), γ-terpinene (7.5%).	1.65%	[44]
*O. syriacum* ssp. *syriacum*	Jordan	Thymol (51.8%), carvacrol (34.4%), *p*-cymene (3.9%).	2.0–2.2%	[53]
	Jordan	Thymol (72.4%), γ-terpinene (7.8%), *p*-cymene (5.4%), carvacrol (3.5%).	2.0–2.2%	
*O. vulgare* L.	Argentina	*p*-Cymene (26.00%), γ-terpinene (21.89%), terpinen-4-ol (16.29%), β-caryophyllene (8.25%).	NR	[54]
	Argentina	Carvacrol (26.70%), *p*-cymene (15.20%), γ-terpinene (15.10%), terpinene (7.50%).	NR	[55]
	Argentina	γ-Terpinene (25.1%), terpinen-4-ol (16.7%), carvacrol (16.2%), α-terpinene (8.54%).	NR	[56]
	Argentina	γ-Terpinene (32.1%), α-terpinene (15.1%), *p*-cymene (8.0%), thymol (8.0%).	NR	[57]
	Argentina	Carvacrol (81.92%), γ-terpinene (4.49%), thymol (3.5%), *p*-cymene (3.07%).	NR	[58]
	Brazil	Carvacrol (73.9%), γ-terpinene (3.6%), thymol (3.0%), β-caryophyllene (2.8%).	NR	[45]
	Chile	cis-β-Terpineol (16.49%), thymol (13.26%), terpinen-4-ol (10.24%), α-terpineol (4.35%).	NR	[59]
	China	Carvacrol (30.73%), thymol (18.81%), *p*-cymene (10.88%), β-caryophyllene (8.21%).	NR	[37]
	China	β-Citronellol (85.3%), citronellol acetate (5.2%), β-citronellal (1.2%).	0.7%	[60]
	China	Thymol (42.9%), citronellol (12.2%), β-caryophyllene (7.8%), *p*-cymen-2-ol (7.5%).	0.3%	[60]
	China	β-Citronellol (75.0%), geraniol (7.7%), citronellol acetate (3.4%).	0.3%	[60]
	China	1,8-Cineole (20.8%), β-caryophyllene (10.2%), eugenol methyl ether (9.8%), citronellol (8.8%).	0.3%	[60]
	China	Caryophyllene oxide (32.9%), β-caryophyllene (17.7%), citronellol (10.2%), germacrene D (9.8%).	0.1%	[60]
	Colombia	Thymol (21.5%), *p*-cymene (21.0%), γ-terpinene (20.3%), α-terpinene (5.9%).	NR	[39]
	Greece	Carvacrol (63.03%), thymol (15.09%), *p*-cymene (10.47%), γ-terpinene (3.43%).	NR	[61]
	India	Carvacrol (35.02–62.81%), *p*-cymene (8.60–46.59%), γ-terpinene (2.49–19.11%).	0.20–1.30%	[62]
	Iran	Carvacrol (29.85%), γ-terpinene (20.94%), α-himachalene (12.17%), β-pinene (11.67%).	0.80%	[63]
	Iran	Carvacrol (23.54%), γ-terpinene (20.50%), thymol (15.41%), germacrene D-4-ol (9.26%).	1.26%	[63]
	Iran	Carvacrol (59.37%), γ-terpinene (18.36%), cedrene (6.65%).	1.66%	[63]
	Iran	Carvacrol (58.51%), humulene (11.46%), γ-terpinene (9.56%).	0.93%	[63]
	Iran	Carvacrol (67.09%), γ-terpinene (7.71%), humulene (7.67%).	1.36%	[63]
	Italy	Cavacrol (65.94%), *p*-cymene (9.33%), γ-terpinene (5.25%), β-caryophyllene (3.72%).	NR	[64]
	Italy	Carvacrol (71.8%), *p*-cymene (11.6%), β-caryophyllene (2.7%), linalool (1.8%).	NR	[65]
	Morocco	Carvacrol (34.0%), γ-terpinene (21.6%), *p*-cymene (9.4%), thymol (3.3%).	2.7%	[66]
	Pakistan	β-Citronellol (72.7%), thymol (7.2%), citronellol acetate (5.9%).	0.3%	[60]
	Poland	Carvacrol (26.38–36.72%), thymol (16.59–25.58%), γ-terpinene (10.06–16.11%), *p*-cymene (6.09–6.76%).	NR	[31]
	Portugal	Carvacrol (14.5%), β-fenchyl alcohol (12.8%), γ-terpinene (11.6%), δ-terpineol (7.5%).	NR	[67]
	Serbia	Sabinene (10.2%), terpinen-4-ol (9.3%), 1,8-cineole (5.8%), γ-terpinene (5.6%).	0.17%	[68]
	Serbia	Carvacrol (64.5%), *p*-cymene (10.9%), γ-terpinene (10.8%), thymol (3.5%).	1.5%	[69]
	Serbia	Carvacrol (64.5%), *p*-cymene (10.9%), γ-terpinene (10.8%), thymol (3.5%).	NR	[70]
	Serbia	Carvacrol (77.6%), *p*-cymene (5.14%), *trans*-β-caryophyllene (2.45%), linalool (2.44%).	NR	[71]
	Spain	Terpinen-4-ol (24.57%), carvacrol (16.09%), thymol (9.03%), γ-terpinene (6.20%).	516 mg/plant	[27]
	USA	Carvacrol (17.9–81.8%), *p*-cymene (2.62–25.7%), γ-terpinene (2.5–19.4%), β-myrcene (0.58–6.06%).	0.114–2.312%	[35]
*O. vulgare* L. ssp. *glandulosum*	Algeria	Thymol (34.2%), carvacrol (30.5%), γ-terpinene (13.4%), *p*-cymene (6.6%).	2.0–2.2%	[53]
	Algeria	Thymol (51.1%), γ-terpinene (14.5%), *p*-cymene (7.5%), carvacrol (6.8%).	2.0–2.2%	[53]
	Tunisia	*p*-Cymene (35.7–46.3%), thymol (18.4–39.1%), γ-terpinene (11.7–24.2%), carvacrol (1.7–15.1%).	2.5–4.6%	[72]
	Tunisia	Thymol (31.8–46.1%), *p*-cymene (11.5–35.7%), γ-terpinene (24.0–27.1%), α-terpinene (1.9–3.2%).	4.3–5.8%	[73]
	Tunisia	Carvacrol (65.01%), *p*-cymene (9.00%), γ-terpinene (4.25%), borneol (3.19%).	1.87–3.42%	[74]
*O. vulgare* L. ssp. *gracile*	Iran	Carvacrol (46.86%), γ-terpinene (14.16%), *p*-cymene (11.63%), carvacrol methyl ether (5.97%).	≈2.0%	[29]
	Turkey	Thymol (7.02–40.04%), carvacrol (8.21–33.21%), γ-terpinene (9.15–27.82%), *p*-cymene (3.07–23.52%).	0.25–0.50%	[75]
*O. vulgare* L. ssp. *hirtum*	Argentina	*trans*-Sabinene hydrate (22.9%), thymol (18.6%), γ-terpinene (7.1%), terpinen-4-ol (6.2%).	NR	[22]
	Argentina	*trans*-Sabinene hydrate (17.9%), thymol (17.1%), terpinen-4-ol (9.5%), γ-terpinene (8.0%).	NR	[22]
	Argentina	γ-Terpinene (13.7%), terpinen-4-ol (11.2%), α-terpinene (9.9%), *trans*-sabinene hydrate (8.3%).	NR	[76]
	Colombia	Carvacrol (90.3%), thymol (3.5%), *p*-cymene (2.7%), γ-terpinene (1.0%).	NR	[39]
	Greece	Carvacrol (70.38%), *p*-cymene (8.17%), γ-terpinene (7.78%), β-myrcene (2.37%).	NR	[48]
	Greece	Carvacrol (90.29%), γ-terpinene (3.09%), *p*-cymene (2.25%), β-caryophyllene (1.81%).	7.77%	[18]
	Greece	Carvacrol (81.28–91.21%), *p*-cymene (1.52–6.40%), γ-terpinene (0.49–4.01%), β-caryophyllene (0.94–2.03%).	4.71–5.00%	[77]
	Greece	Carvacrol (56.46–82.70%), *p*-cymene (9.54–21.40%), β-disavolene (1.09–3.06%).	0.63–4.25%	[78]
	Hungary	Carvacrol (82.75%), *p*-cymene (6.58%), γ-terpinene (5.78%).	4.46%	[30]
	Italy	terpinen-4-ol (13.27–17.51%), γ-terpinene (14.58–14.95%), carvacrol (12.31–14.58%), *p*-cymene (8.43–10.07%).	0.063–0.165%	[79]
	Italy	Thymol (37.9%), γ-terpinene (24.5%), *p*-cymene (16.3%), α-terpinene (4.3%).	NR	[80]
	Italy	γ-Terpinene (29.41%), thymol (26.86%), *p*-cymene (8.20%), α-terpinene (5.93%).	5.4%	[81]
	Italy	Thymol (37.22%), γ-terpinene (26.37%), *p*-cymene (6.83%), α-terpinene (4.02%).	2.4%	[81]
	Italy	Thymol (36.46%), γ-terpinene (20.77%), *p*-cymene (8.31%), carvacrol methyl ether (6.21%).	3.6%	[81]
	Italy	Thymol (30.25%), γ-terpinene (25.89%), *p*-cymene (7.62%), carvacrol methyl ether (5.63%).	4.2%	[81]
	Italy	Thymol y carvacrol (65.3–84.7%), linalool (0.1–2.6%), carvacrol methyl ether (0.4–1.9%).	1.0–2.7%	[82]
	Italy	Thymol (18.16–56.37%), γ-terpinene (12.70–32.70%), *p*-cymene (8.22–10.30%).	1.7–4.5%	[28]
	Lithuania	Carvacrol (72.4–88.2%), γ-terpinene (4.1–8.7%), *p*-cymene (2.0–3.2%), β-caryophyllene (0.9–3.0%).	35.50–325.45 dm^3^/ha	[24]
	Serbia	Carvacrol (74.65%), *p*-cymene (5.87%), γ-terpinene (5.04%), *trans*-β-caryophyllene (1.76%).	1.34%	[36]
	Turkey	Linalool (96.31%), β-caryophyllene (1.27%).	7.31%	[83]
	Turkey	Carvacrol (80.09%), γ-terpinene (12.01%), *p*-cymene (1.72%), α-terpinene (1.58%).	5.9 ± 0.02%	[21]
*O. vulgare* L. ssp. *virens*	Argentina	*trans*-Sabinene hydrate (27.77%), thymol (26.1%), γ-terpinene (5.9%), α-terpinene (4.17%).	2.17 ± 0.32 mg/g dw	[43]
	Iran	(*Z*)-α-Bisabolene (39.17%), sabinene (11.52%), carvacrol (5.23%), β-bisabolene (4.24%).	≈0.3%	[29]
	Portugal	α-Terpineol (0.1–65.1%), γ-terpinene (0.3–34.25), linalool (2.0–27.4%), carvacrol (0–34.2%), *E*-caryophyllene (2.4–11.0%).	0.8–1.2%	[84]
*O. vulgare* L. ssp. *vulgare*	Argentina	*trans*-Sabinene hydrate (23.4–27.2%), thymol (14.4–17.2%), terpinen-4-ol (7.8–11.0%), γ-terpinene (7.3–9.8%).	NR	[22]
	Argentina	*trans*-Sabinene hydrate (32.47%), thymol (20.5%), γ-terpinene (15.47%), terpinen-4-ol (5.03%).	1.97 ± 0.22 mg/g dw	[43]
	Iran	Thymol (37.13%), γ-terpinene (9.67%), carvacrol (9.57%), carvacrol methyl ether (6.88%).	0.5%	[85]
	Italy	Spathulenol (18.6%), carvacrol (11.7%), β-caryophyllene (8.8%), terpinen-4-ol (5.6%).	0.13%	[26]
	Italy	Carvacrol (14.3%), spathulenol (9.4%), β-caryophyllene (5.3%), terpinen-4-ol (5.0%).	0.18%	[26]
	Lithuania	Sabinene (6.6–28.2%), β-caryophyllene (7.3–15.5%), *E*-β-ocimene (4.4–15.1%), *allo*-ocimene (7.7–12.1%).	3.08–36.65 dm^3^/ha	[24]
	Turkey	Thymol (58.31%), carvacrol (16.11%), *p*-cymene (13.45%), γ-terpinene (4.64%).	5.09%	[83]
	Poland	Sabinene (10.85–25.46%), *Z*-(β)-ocimene (9.10–16.33%), germacrene D (9.36–15.34%), *E*-caryophyllene (9.38–12.87%).	0.66–0.86%	[86]

* dry weight (dw); NR: not reported.

**Table 2:** Summarization of the biological activities of essential oils from oregano.

Oregano Species	Biological Activity	Effect	Reference
*H. patens*	Anti-inflammatory	Reduction on the levels of NO and ROS produced in murine macrophage cells.	[16]
*L. palmeri*	Anti-inflammatory	Inhibition on the production of ROS and NO by LPS-stimulated RAW 264.7 macrophages	[16]
*L. graveolens*	Antioxidant	Radical scavenging activity against DPPH	[90]
	Anti-inflammatory	Reduction on the levels of NO and ROS produced in LPS-stimulated murine macrophage cells	[16]
*O. acutidens*	Antioxidant	Showed scavenging activity against DPPH radical	[42]
*O. compactum*	Antioxidant	ABTS radical-scavenging activity	[111]
	Cytotoxic	Nontoxic when used in MCF-7 cells	[111]
*O. dictamnus*	Antioxidant	Ferric reducing/antioxidant power	[117]
	Antiproliferative	Inhibit colon carcinoma (LoVo) and hepatocarcinoma (HepG2) cell proliferation	[117]
*O. ehrenbergii*	Antioxidant	DPPH radical-scavenging activity	[107]
*O. glandulosum*	Antioxidant	Showed antiradical activity	[72,73]
*O. heracleoticum*	Anti-inflammatory	Inhibition of NO production	[100]
*O. libanoticum*	Antioxidant	Ferric reducing/antioxidant power	[117]
	Antiproliferative	Inhibit HepG2 cell proliferation	[117]
*O. majorana*	Anti-inflammatory	Reduction in the secretion of inflammatory cytokines (TNF-α, IL-1β and IL-6) in THP-1 cells	[124]
	Anti-genotoxic	Reduces the chromosomal aberration in bone marrow cells of rats	[162]
	Cytotoxic	Inhibit cell viability of human breast (MCF-7) and prostate (LNCaP) cancer cell lines.	[163]
*O. microphyllum*	Antioxidant	Showed ferric reducing power	[117]
*O. minutiflorum*	Antioxidant	Retard lipidic oxidation	[116]
*O. onites*	Antioxidant	Showed free radical scavenging against DPPH radical	[50]
	Anti-angiogenic	Blocks in vitro tube formation	[159]
*O. rotundifolium*	Anti-genotoxic	Reduces the effect of Aflatoxin B_1_ (AFB_1_) in human peripheral lymphocytes	[161]
*O. syriacum*	Antioxidant	DPPH radical-scavenging activity	[107]
*O. virens*	Antioxidant	Showed scavenging activity against DPPH radical	[118]
*O. vulgare* subsp. *hirtum*	Antioxidant	Total reducing capacity (Folin-Ciocalteu method), radical-scavenging activity in the UV radiation-induced peroxidation in liposomal membranes	[101]
	Antioxidant	Reduces 8-hydroxy-deoxyguanosine and thiobarbituric acid reactive substances.	[102]
	Antioxidant	DPPH and ABTS radical-scavenging activity	[80,83,114,115]
	Antiproliferative	Inhibit human lung adenocarcinoma epithelial (A549) cell proliferation	[76]
	Cytotoxic	Decrease cell viability in a concentration-dependent manner on human keratinocyte (HaCaT) and lung cancer (A549) cell lines	[80]
	Hypoglycemic	α-Amylase and α-glucosidase inhibitory activity	[83]
*O. vulgare* subsp. *vulgare*	Antioxidant	Radical scavenging activity (DPPH, ABTS and FRAP assays). Total reducing capacity (Folin-Ciocalteu method)	[37,55,56,57,83,110,112,85]
	Antioxidant	Prevent autoxidation of polyunsaturated fatty acid esters	[106]
	Anti-inflammatory	Reduced synthesis of TNF-α, IL-1β, and IL-6 cytokines. Increased synthesis of cytokine IL-10	[140]
	Anti-inflammatory	Inhibition of the levels of inflammatory biomarkers (MCP-1, VCAM-1 and ICAM-1) on activated-primary human neonatal fibroblasts	[125]
	Antiproliferative	Inhibit human breast adenocarcinoma (MCF-7) and human colon adenocarcinoma (HT-29) cell proliferation	[157]
	Antitumor	Decrease the sizes of tumors in disease mice	[158]
	Hypoglycemic	Inhibits α-amylase and α-glucosidase activity	[83,153]
